# Impact of the geriatric nutritional risk index on long-term outcomes in patients undergoing hemodialysis: a meta-analysis of observational studies

**DOI:** 10.3389/fnut.2024.1346870

**Published:** 2024-03-21

**Authors:** Kuo-Chuan Hung, Chia-Li Kao, Chih-Wei Hsu, Chia-Hung Yu, Chien-Ming Lin, Hsiao-Tien Chen, Ying-Jen Chang, Shu-Wei Liao, I-Wen Chen

**Affiliations:** ^1^Department of Anesthesiology, Chi Mei Medical Center, Tainan, Taiwan; ^2^Department of Anesthesiology, E-Da Hospital, I-Shou University, Kaohsiung, Taiwan; ^3^Department of Psychiatry, Kaohsiung Chang Gung Memorial Hospital and Chang Gung University College of Medicine, Kaohsiung, Taiwan; ^4^Department of Chinese Medicine, Chi Mei Medical Center, Tainan, Taiwan; ^5^Department of Anesthesiology, Chi Mei Medical Center, Liouying, Tainan, Taiwan

**Keywords:** geriatric nutritional risk index, hemodialysis, overall mortality, meta-analysis, renal failure

## Abstract

**Background:**

This meta-analysis aimed to synthesize current evidence on the association between the Geriatric Nutritional Risk Index (GNRI) and long-term outcomes in patients undergoing hemodialysis.

**Methods:**

Electronic databases were systematically searched for relevant studies that investigated the association between GNRI and long-term outcomes in hemodialysis patients until November 2023. The primary outcome was the association between the GNRI (i.e., low versus high) and overall mortality risk, while the secondary outcome was the relationship between the GNRI and cardiovascular mortality risk.

**Results:**

Thirty cohort studies involving 55,864 patients were included. A low GNRI was found to be significantly associated with increased overall mortality (hazard ratio [HR]: 2.42, 95% confidence interval [CIs]: 2.10–2.79, *p* < 0.00001, *I*^2^ = 65%). Each unit increase in GNRI corresponded to a 5% reduction in mortality risk (HR: 0.95, 95% CI: 0.93–0.96, *p* < 0.00001, *I*^2^ = 79%). The association remained consistent across Asian (HR = 2.45, 95% CI: 2.08–2.88, *p* < 0.00001, *I*^2^ = 70%) and non-Asian subgroups (HR = 2.3, 95% CI: 1.72–3.06, *p* < 0.00001, *I*^2^ = 23%). Meta-regression analysis of patient age (coefficient: −0.002; *p* = 0.896), male proportion (coefficient: 0.002; *p* = 0.875), percentage of diabetes mellitus (coefficient: −0.003; *p* = 0.605), and follow-up duration (coefficient: −0.003; *p* = 0.431) revealed that these moderator variables did not significantly influence the association between GNRI and overall mortality risk. Cardiovascular mortality risk also increased with low GNRI (HR, 1.93; 95%CI: 1.51–2.45, *p* < 0.00001; *I*^2^ = 2%). Similarly, an inverse association was observed between the GNRI values and cardiovascular mortality risk (HR, 0.94; 95% CI: 0.91–0.97; *p* < 0.0001; *I*^2^ = 65%) (per unit increase).

**Conclusion:**

The GNRI is a simple nutritional screening tool that can be used to effectively stratify patients undergoing hemodialysis globally. Further studies are warranted to determine whether nutrition optimization based on the GNRI improves long-term outcomes.

**Systematic review registration:**

https://www.crd.york.ac.uk/prospero/, CRD42023483729.

## 1 Introduction

End-stage renal disease (ESRD) is a global public health problem, with increasing prevalence rates worldwide in conjunction with population aging and increasing incidences of diabetes mellitus (DM), hypertension, and chronic kidney disease (1–4). The average number of new ESRD diagnoses worldwide is 144 individuals per million populations (5). However, the incidence of ESRD varies significantly across countries. In China, the estimated number of patients undergoing hemodialysis is expected to increase to 629.67 individuals per million populations in 2025 (6). Hemodialysis is the predominant form of renal replacement therapy in patients with ESRD (7). Compared with the general population, patients with ESRD undergoing hemodialysis have multiple comorbidities, impaired quality of life, malnutrition, and dramatically increased risks of cardiovascular events and premature mortality (8–10). The first-year mortality rate in elderly patients undergoing dialysis was reported to range from 30 to 54.5% (11). Protein-energy wasting is a multifaceted metabolic condition characterized by diminished mass of muscle and adipose tissue, frequently accompanied by reduced appetite and nutritional status (12). It is a common issue among patients undergoing hemodialysis, with its prevalence varying between 28 and 54% in patients undergoing regular dialysis treatment (9, 13–15). Current evidence suggests that protein energy wasting or malnutrition is a potential predictor of morbidity and mortality in this population (12, 16).

The Geriatric Nutritional Risk Index (GNRI) was developed as a simplified nutritional screening tool for assessing the risk of nutrition-related complications and nutritional status in patients undergoing maintenance hemodialysis (17–20). This tool is calculated based on serum albumin levels, body weight, and ideal body weight (18, 21). A previous meta-analysis of 10,739 patients from 19 cohort studies published between 2010 and 2018 revealed that GNRI was significantly associated with overall mortality and cardiovascular events in hemodialysis patients (22). However, the findings from the meta-analysis might not fully represent the long-term risk associated with clinical outcomes, as the associations were assessed at a single point in time—for instance, displaying results as odds ratios (ORs)—without considering the potential effects of interactions over time (22). Additional evidence is warranted to elucidate the association between the GNRI and long-term mortality risk in hemodialysis patients through time-to-event analyses. Recently, there has been a growing trend in the number of studies conducted to examine the association between the GNRI and long-term mortality risk in patients on maintenance hemodialysis, providing an opportunity to update the existing pool of knowledge (23–33). This meta-analysis aimed to synthesize the current evidence and provide a quantitative estimate of the association between GNRI and mortality risk in hemodialysis patients.

## 2 Materials and methods

The study protocol was registered with PROSPERO under registration number CRD42023483729, and the meta-analysis was executed in compliance with the PRISMA guidelines (34).

### 2.1 Data sources and search strategies

To achieve a comprehensive review of the relevant literature, we rigorously searched multiple electronic databases, including MEDLINE, Embase, Cochrane Library, and Google Scholar, to identify observational studies that investigated the association between the GNRI and long-term mortality risk in hemodialysis patients. The search period spanned from the inception of these databases until November 17, 2023. Our search strategy involved a combination of terms associated with “geriatric nutritional risk index,” “mortality,” and “hemodialysis,” and no language restrictions were imposed. In addition to electronic databases, we explored the reference lists of the included studies or relevant review articles for any additional pertinent publications. Table 1 presents a summary of the detailed search strategy for one of the databases (i.e., MEDLINE).

**Table 1:** Search strategy for MEDLINE.

#1	(“Dialysis, Renal” or “Hemodialysis” or “Hemodialyses” or “Extracorporeal Dialyses” or “Extracorporeal Dialysis” or “Kidney Dialysis” or “Renal Dialysis” or “Blood Dialysis” or “Artificial Kidney Treatment” or “End-Stage Renal Disease “or “End-Stage Kidney Disease” or “Chronic Kidney Failure” or “End-Stage Renal Failure” or “Renal Failure, Chronic” or “Chronic Renal Failure” or “ESRD”).mp.
#2	exp “Renal Dialysis”/ or exp. “Kidney Failure, Chronic”/
#3	(“geriatric nutritional risk index” or “GNRI”).mp.
#4	(“Mortality” or “Cardiovascular mortality” or “Survival” or “Survival Analysis” or “Kaplan–Meier Survival Curves”).mp.
#5	exp “Mortality”/ or exp. “Survival”/ or exp. “Kaplan–Meier Estimate”/
#6	(#1 or #2) and #3 and (#4 or #5)

### 2.2 Study selection process

To ensure a systematic approach in selecting relevant studies, a two-step screening process was used. First, two independent reviewers screened the titles and abstracts of retrieved records to assess their potential eligibility. Second, full-text assessments of the selected records were conducted to determine final inclusion in the study. Any discrepancies or disagreements between the reviewers were promptly addressed by consulting a third reviewer, thereby ensuring uniformity and minimizing the potential for bias. The study selection adhered to a predefined protocol and specific selection criteria, ensuring transparency and replicability in the study selection process.

### 2.3 Inclusion and exclusion criteria

The following inclusion criteria were used for study selection: (1) studies involving adult patients undergoing maintenance hemodialysis therapy regardless of hemodialysis vintage; (2) studies assessing or reporting on the GNRI as a prognostic factor; (3) studies reporting on the association between the GNRI and time-dependent variables, including overall and cardiovascular mortality risk; and (4) cohort studies. The exclusion criteria were as follows: (1) case reports, reviews, editorials, and studies that did not provide relevant outcome data; (2) cross-sectional studies (excluded due to the lack of time-dependent variables); and (3) studies focused on patients undergoing peritoneal dialysis or those suffering from acute kidney injury.

### 2.4 Data collection

Relevant data were extracted independently by two team members using a standardized form, including study characteristics, participant demographics (e.g., age and male proportion), sample size, hemodialysis duration, percentage of patients with DM, GNRI cutoff values (low/high), follow-up duration, country in which the study was conducted, and mortality outcomes. For mortality outcomes, we only collected time-dependent variables (i.e., HR). In cases where information was missing, we contacted the corresponding authors of the article to request necessary details.

### 2.5 Outcomes and definition

The primary outcome was overall mortality, defined as death from any cause, while the secondary outcome was cardiovascular mortality. Low GNRI was the main exposure variable, as defined by the individual study. We conducted subgroup analyses to explore the impact of the geographical location of the study populations (i.e., Asian and non-Asian) on the primary outcome.

### 2.6 Quality assessment of included studies

The quality of the included studies was assessed using the Newcastle–Ottawa Scale, which consists of three main components: selection, comparability, and outcome of interest. Studies were awarded stars in each category, with more stars indicating a higher quality. Each study was awarded a maximum of nine stars, with studies receiving six or more stars deemed high quality. This threshold was set to distinguish studies with a lower risk of bias and robust methodological approach. Disagreements were resolved by consensus or consultation with a third reviewer.

### 2.7 Statistical analysis

Statistical analyses were conducted using Comprehensive Meta-Analysis software (Version 4, Biostat, Englewood, NJ). Owing to the anticipated clinical and methodological heterogeneity among the included studies, a random-effects model was used. Heterogeneity among studies was quantified using the *I*^2^ statistic, which describes the percentage of total variation across studies caused by heterogeneity rather than by chance. An *I*^2^ value exceeding 50% indicated considerable heterogeneity among studies. Sensitivity analyses were conducted to evaluate the impact of each study on combined effect size. Furthermore, funnel plots were created to assess publication bias for outcomes reported in more than 10 studies. A meta-regression analysis was conducted to explore the potential sources of heterogeneity and assess the impact of the moderator variables on the effect size. The variables included age, male proportion, percentage of patients with DM, and follow-up duration. These variables were selected based on their clinical relevance and likelihood of influencing the association between the GNRI and long-term mortality. The statistical significance of the pooled estimates and meta-regression coefficients was set at *p <* 0.05.

## 3 Results

### 3.1 Study selection and characteristics of studies

**Figure 1:**
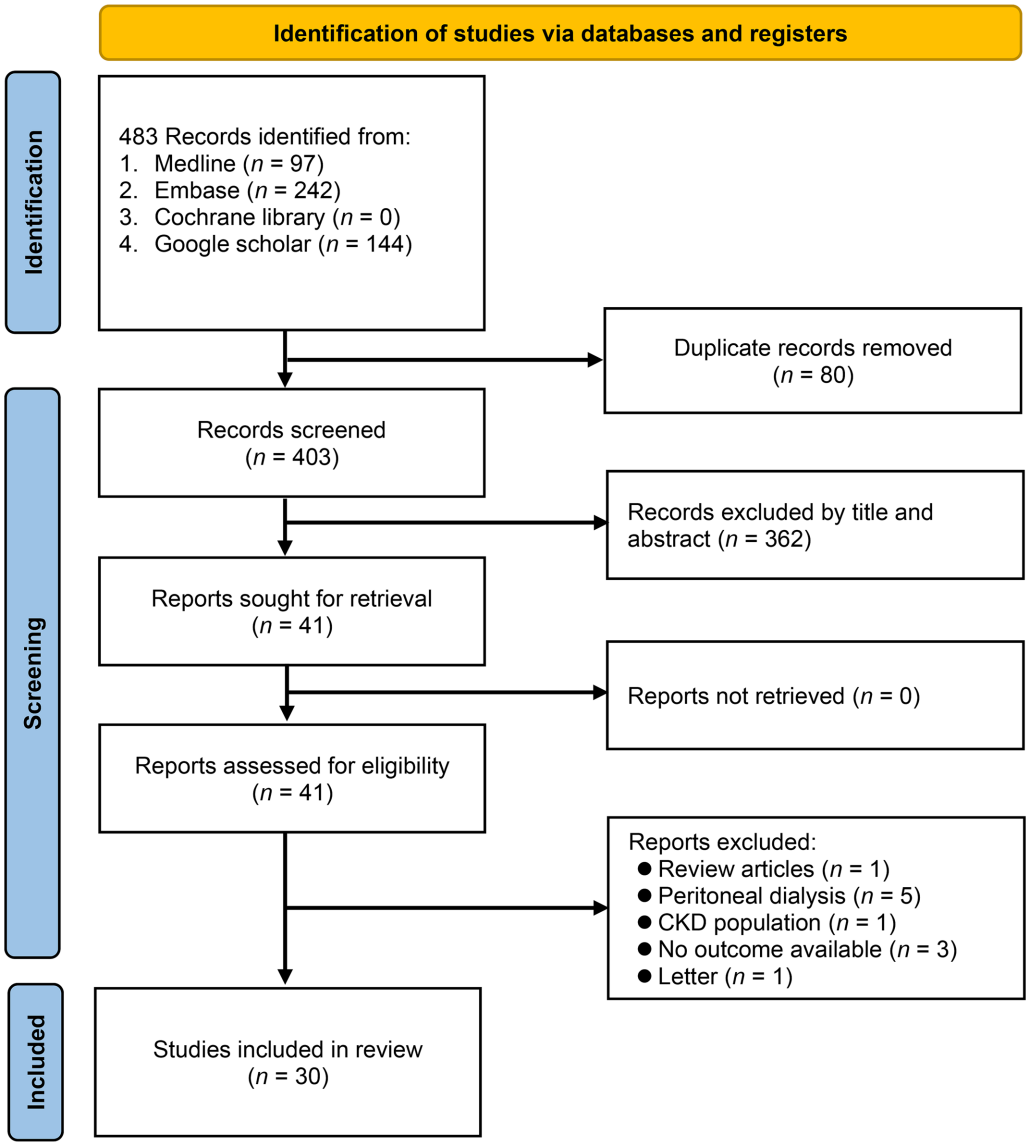
The flowchart outlining the study selection process for current meta-analysis. CKD, chronic kidney disease.

The selection process for the meta-analysis is illustrated in Figure 1. Database searches, including PubMed, Embase, Cochrane Library, and Google Scholar, initially yielded 403 records. After removing 80 duplicates and 362 records based on titles and abstracts, 41 records were retrieved for full-text review. All of these were assessed for eligibility, and various reports were excluded for reasons such as review articles or irrelevant to the study population. Ultimately, 30 studies were deemed suitable and included in the meta-analysis (23–33, 35–53).

Table 2 presents data from 30 studies published between 2010 and 2023 that examined the association between GNRI and mortality in hemodialysis patients. These studies were conducted globally across countries, including Turkey, Japan, Korea, China, Taiwan, Israel, Italy, the Netherlands, Iran, and Brazil. The mean or median age of the patients ranged from 49 to 72 years, with the percentage of males ranging from 42.5 to 69.4%. The sample sizes varied significantly across studies, with a minimum of 75 patients and a maximum of 34,933 patients, accumulating in a combined total of 55,864 patients. The hemodialysis vintage varied from 6.4 to 110.4 months in 26 studies, while four studies did not provide relevant information. The percentage of patients with DM as a comorbidity ranged from 15 to 61.5%. The mean GNRI scores ranged from 91.2 to 109.2. The follow-up period for mortality analysis ranged from 12 to 120 months. All 30 studies assessed in the analysis were considered to be of high quality with a low risk of bias, as each scored seven or higher on the Newcastle–Ottawa Scale.

**Table 2:** Characteristics of 30 studies involving 55,864 hemodialysis patients.

Studies	Age (years)†	Male (%)	*n*	Hemodialysis (m)	DM (%)	GNRI	Cut-off (low/high)	Follow-up (m)	Country	NOS
Ayerden Ebinc 2022	57	50.3	169	33	15	105.9 ± 16.6	104.2	12	Turkey	7
Beberashvili 2013	64.8	57	75	41	46.7	na	113	46.8	Israel	7
Beberashvili 2016	67.4	62	352	23.0	57	109.2 ± 12.4	na§	30	Israel	8
Chen 2019	53.9	57	1,025	24.5	27	95.0 ± 6.9	82/98	28.1	China	7
Cho 2022	62	62	2,313	na	54	96.9 ± 6.91	92	37.2	Korea	8
de Roij van Zuijdewijn 2015	63.3	61	489	24	24	na	na⁋	35.6	Netherlands	8
Edalat-Nejad 2015	60	53	145	60	33	102.6 ± 5.5	100	18	Iran	7
Fujioka 2022	68.3	54	183	97	41	91.2 ± 10.9	91.6	60	Japan	8
Ishii 2017	64	63	973	24	48	94.1 ± 8.8	91.2	96	Japan	9
Jung 2014	55.7	42.5	120	65.2	23	99.8 ± 9.9	90	120	Korea	8
Kim 2023	60.2	58.8	34,933	67.2	61.5	98.7	90.8	53.7	Korea	9
Kobayashi 2010	60	59.8	490	88	25	98.0 ± 6.0	na§	60	Japan	9
Komatsu 2015	65.4	64.2	332	67.2	47.9	96.8 ± 8.9	na§	36	Japan	8
Lin 2020	56.5	47	151	na	41	101 ± 6.3	94/98	60	Taiwan	8
Machiba 2022	61.0	62.9	518	110.4	21.2	95.2 (90.8–98.3)	92.3/96.8	60	Japan	9
Matsukuma 2019	68.9	49.5	3,436	58.8	26.9	na	90.8/100.2	48	Japan	8
Naito 2022	65	67	499	64	37	95 (90–100)	92	60	Japan	9
Panichi 2014	65.7	60.7	753	70.4	18.8	93.4 ± 10.7	90.6	84	Italy	9
Park 2012	56.2	42.5	120	54.9	48.3	100.4 ± 9.0	na§	90	Korea	8
Ren 2020	50.2	55.4	1,804	31.8	18.7	92.96 ± 9.15	82/98	33.7	China	8
Rodrigues 2019	69.4	65	173	34.7	38	na	91.2	23.6	Brazil	7
Takahashi 2014	64	66.9	1,568	na	52	na	84.9/97.3	63	Japan	9
Takahashi 2017	64	55.3	409	8	31.8	93.2 ± 5.6	na§	52	Japan.	8
Tsai 2014	49	48.7	318	na	26.7	na	92	54	Taiwan	8
Tsai 2016	72	51.9	104	65	36.5	na	92	38.5	Taiwan	7
Yajima 2022	63.8	66.5	263	18	42.6	93.1 ± 7.6	91.2	37.2	Japan	8
Yajima 2020	63.6	69.4	229	6.4	45.8	94.0 ± 7.0.	91.2	44.4	Japan	8
Yajima 2021	63.4	68.3	180	7.2	46.7	94.5 ± 6.9	91.2	55.2	Japan	7
Yajima 2019	65.1	69.2	234	9.6	45.3	93.5 ± 6.5	94.5	33.6	Japan	8
Yamada 2020	66	65	3,536	50.4	39.9	na	89.3/98.8	26.4	Japan	8

†Mean or median; na, not available; ⁋The GNRI value was classified into four distinct groups; §The relationship between GNRI and mortality was examined by treating GNRI as a continuous variable. NOS, Newcastle–Ottawa Scale; m, months.

### 3.2 Outcomes

#### 3.2.1 Primary outcomes

Of the 30 included studies, 25 provided categorical GNRI data (low versus high GNRI groups), allowing them to be pooled for the primary meta-analysis. The other five studies only provided continuous GNRI data and did not have categorical cutoff values. These five studies were not excluded, as we utilized them in a separate analysis to examine the impact of the GNRI on mortality risk when treating the GNRI as a continuous variable.

**Figure 2:**
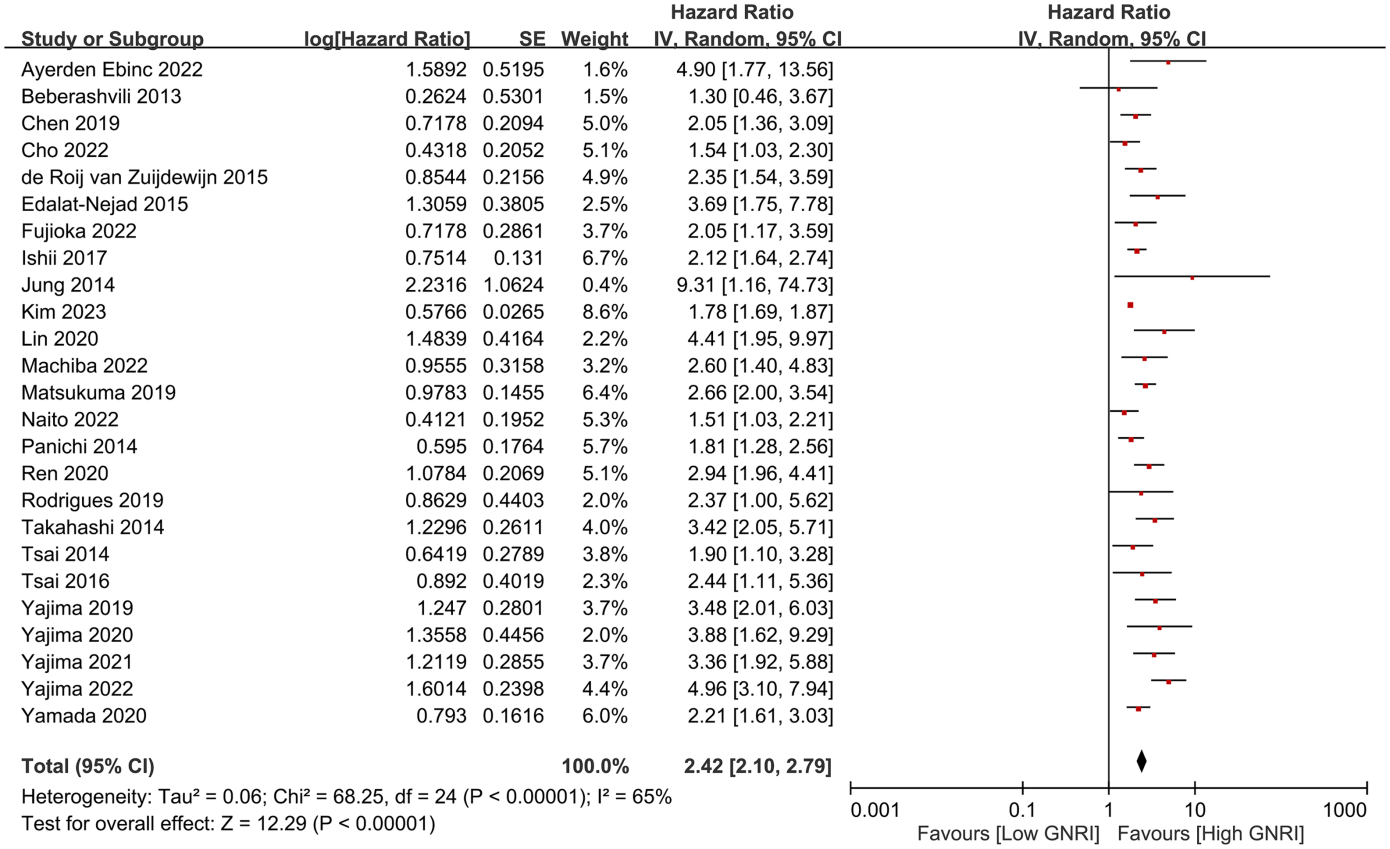
Forest plot showing the association between the Geriatric Nutritional Risk Index (GNRI) and overall mortality. CI, confidence intervals.

**Figure 3:**
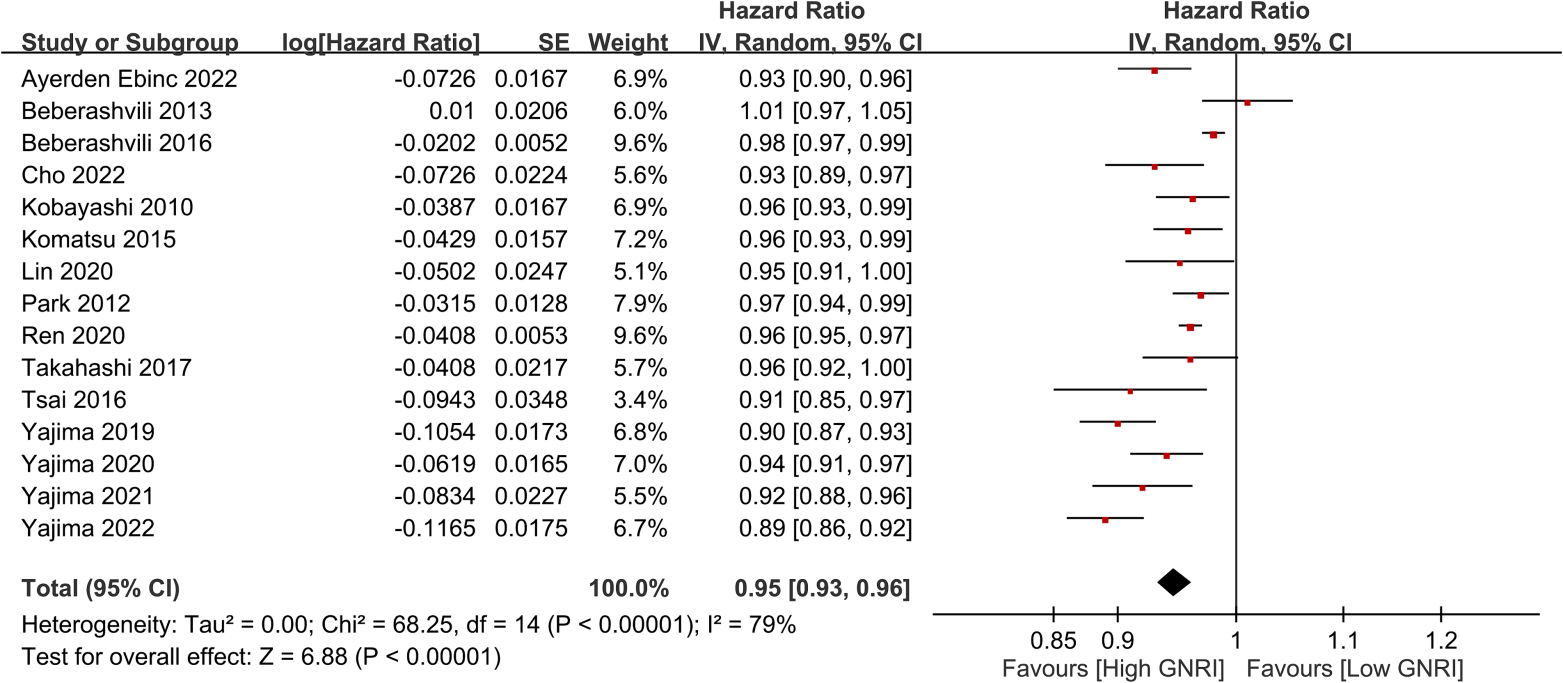
Forest plot showing that with each unit increase in Geriatric Nutritional Risk Index (GNRI), there was a corresponding decrease in overall mortality risk. CI, confidence intervals.

**Figure 4:**
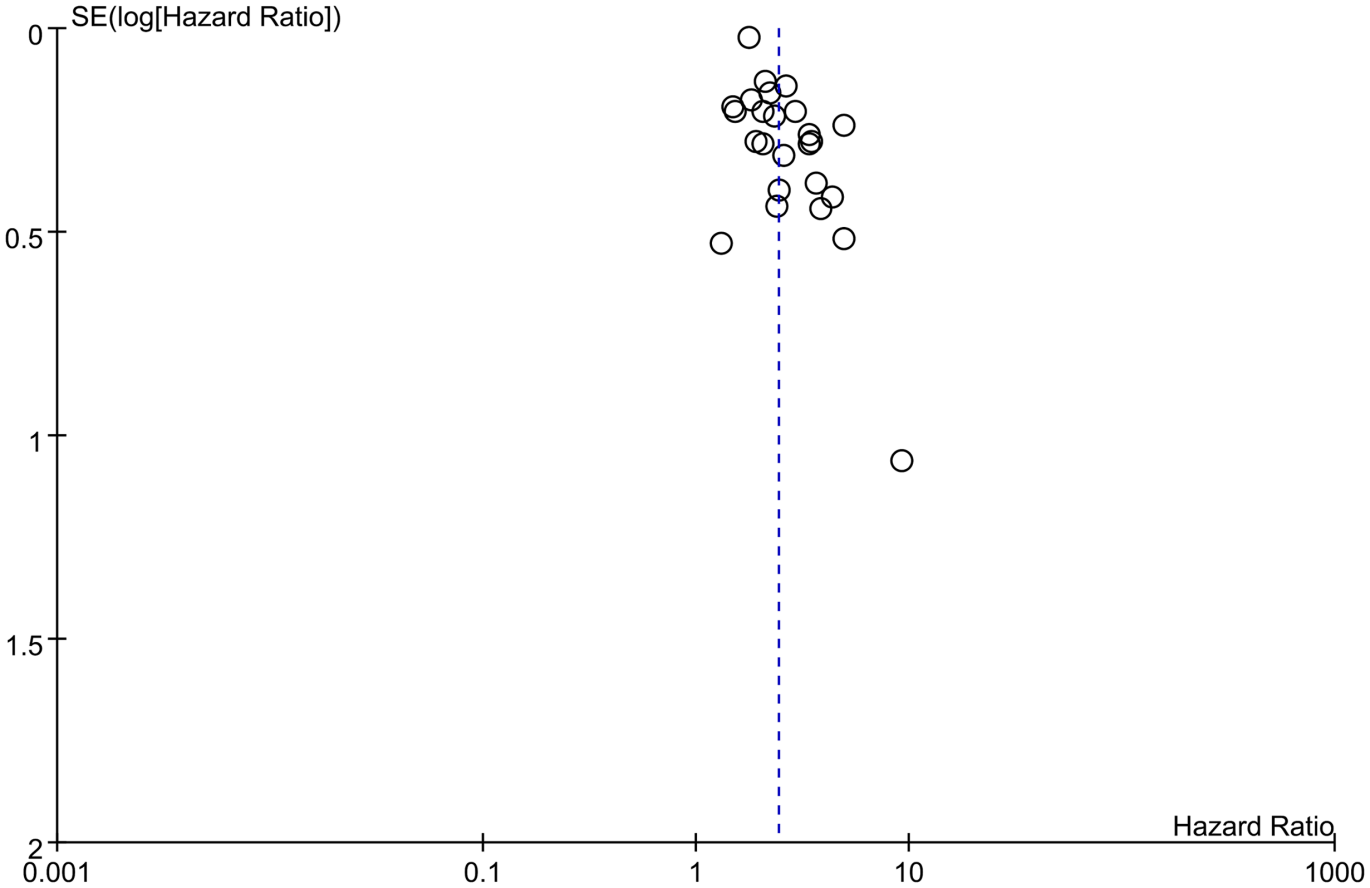
Funnel plot showing the publication bias in studies examining the association between the geriatric nutritional risk index (GNRI) and overall mortality.

In our meta-analysis on 25 studies, we found a significant association between lower GNRI and increased long-term mortality risk, with a combined HR of 2.42 (95% CI, 2.10–2.79, *p* < 0.00001), indicating a more-than-twofold-higher risk of mortality (Figure 2) (23–33, 35, 36, 38–42, 45, 46, 48, 50–53). Sensitivity analysis revealed that this association was evident across multiple studies despite moderate to high heterogeneity (*I*^2^ = 65%), reinforcing the value of the GNRI as a prognostic indicator for this patient population. Additional analysis revealed that with each unit increase in GNRI, there was a corresponding decrease in the overall mortality risk (HR: 0.95, 95% CI: 0.93–0.96, *p* < 0.00001, *I*^2^ = 79%), suggesting an inverse association (Figure 3) (23–25, 27, 28, 32, 35–37, 43, 44, 47, 49, 52, 53). The funnel plot shows a largely symmetrical spread of studies around the combined effect estimate, suggesting minimal publication bias (Figure 4) (23–33, 35, 36, 38–42, 45, 46, 48, 50–53).

**Figure 5:**
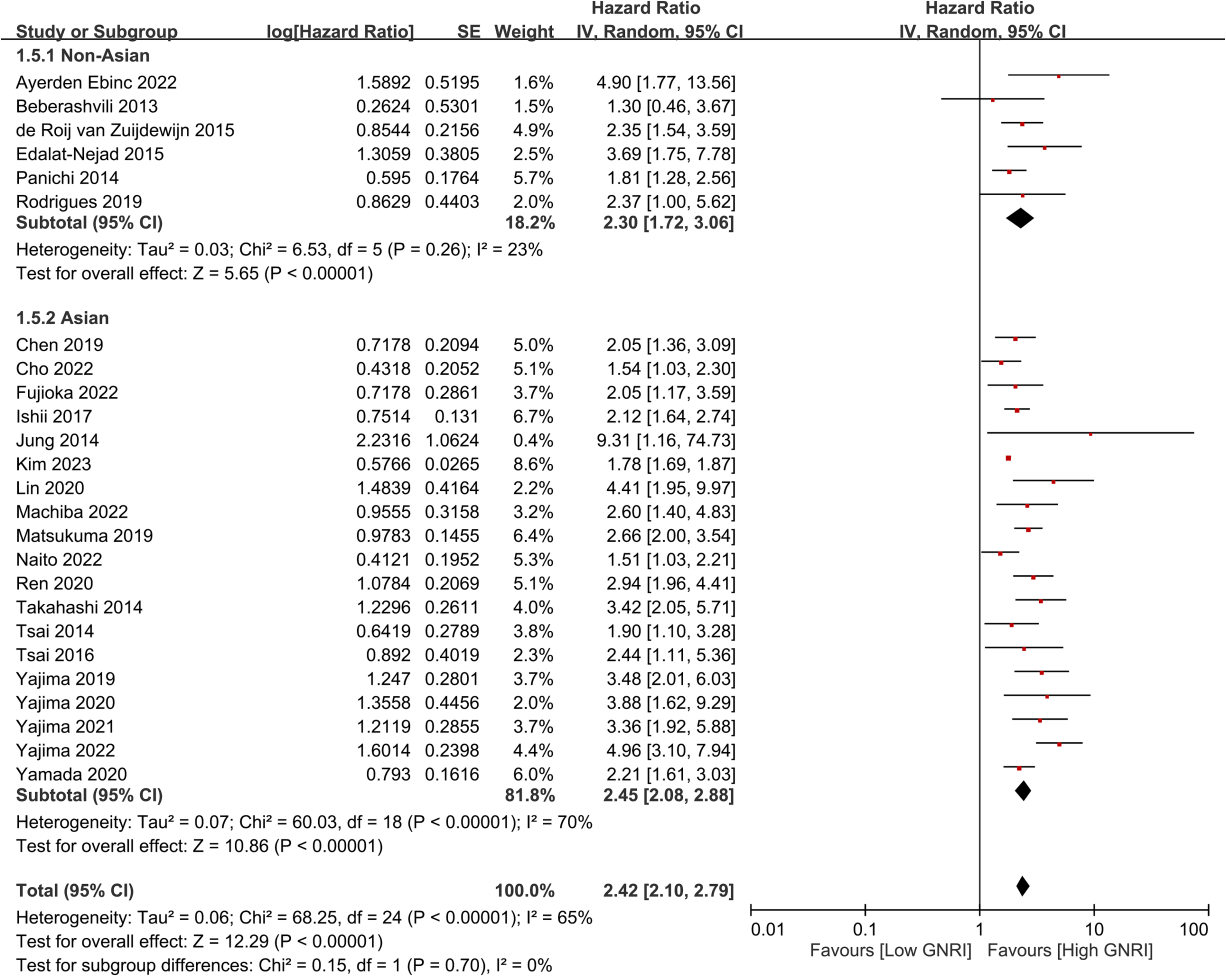
Subgroup analysis showing the association between the geriatric nutritional risk index (GNRI) and overall mortality across different ethnic populations. CI, confidence intervals.

Subgroup analyses revealed a significant association between lower GNRI and increased overall mortality risk (HR = 2.3, 95% CI: 1.72–3.06, *p* < 0.00001, *I*^2^ = 23%) in non-Asian population (Figure 5) (23–33, 35, 36, 38–42, 45, 46, 48, 50–53). For Asian studies, this association was also significant and slightly stronger (HR = 2.45, 95% CI: 2.08–2.88, *p* < 0.00001), albeit with substantial heterogeneity (*I*^2^ = 70%) (Figure 5) (23–33, 35, 36, 38–42, 45, 46, 48, 50–53). This finding suggests that the GNRI is a consistent predictor of mortality across different ethnic populations.

**Figure 6:**
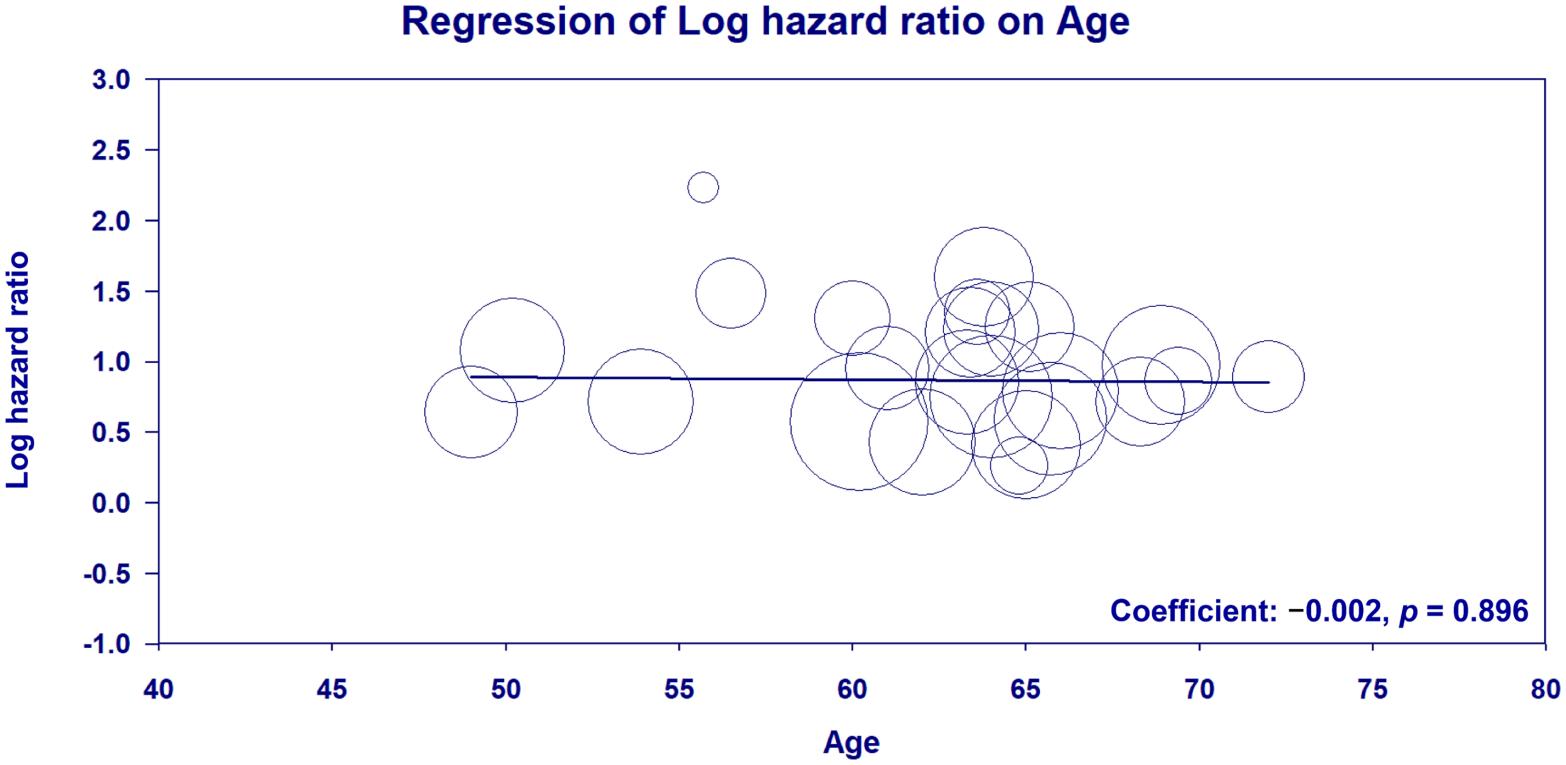
Meta-regression plot analyzing the impact of the age on the effect size on the association between the geriatric nutritional risk index (GNRI) and overall mortality.

**Figure 7:**
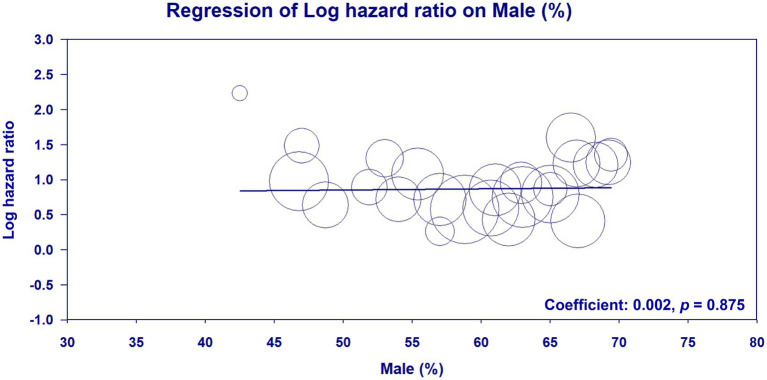
Meta-regression plot analyzing the impact of the male proportion on the effect size of the association between the geriatric nutritional risk index (GNRI) and overall mortality.

**Figure 8:**
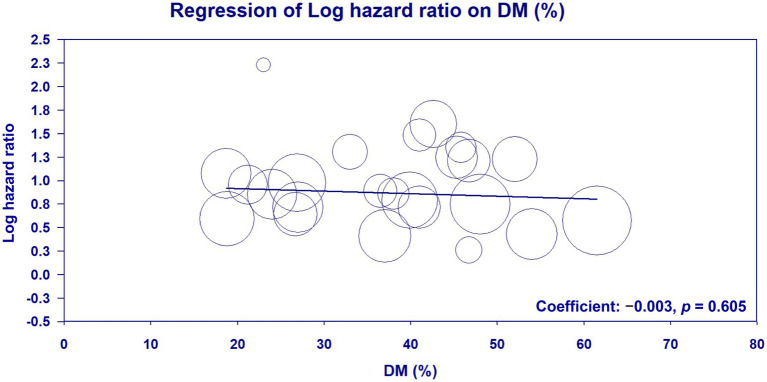
Meta-regression plot analyzing the impact of percentage of patients with diabetes mellitus (DM) on the effect size on the association between the geriatric nutritional risk index (GNRI) and overall mortality.

**Figure 9:**
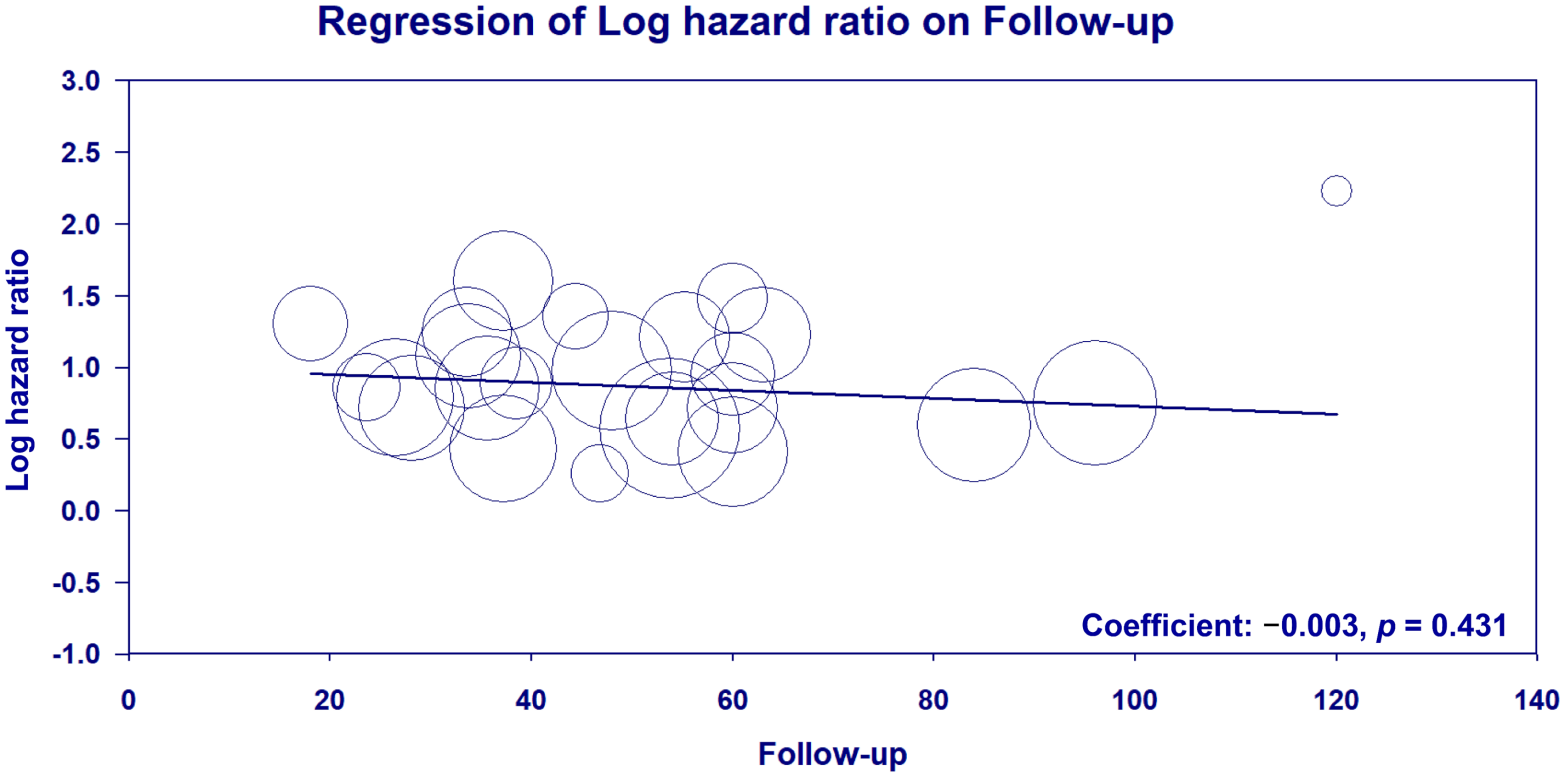
Meta-regression plot analyzing the impact of follow-up duration on the effect size on the association between the geriatric nutritional risk index (GNRI) and overall mortality.

Meta-regression analysis of patient age (coefficient: −0.002; *p* = 0.896) (Figure 6) (23–33, 35, 36, 38–42, 45, 46, 48, 50–53), male proportion (coefficient: 0.002; *p* = 0.875) (Figure 7) (23–33, 35, 36, 38–42, 45, 46, 48, 50–53), percentage of patients with DM (coefficient: −0.003; *p* = 0.605) (Figure 8) (23–33, 35, 36, 38–42, 45, 46, 48, 50–53), and follow-up duration (coefficient: −0.003; *p* = 0.431) (Figure 9) (23–33, 35, 36, 38–42, 45, 46, 48, 50–53) revealed that these moderator variables did not significantly impact the association between GNRI and mortality outcomes.

#### 3.2.2 Secondary outcomes

**Figure 10:**
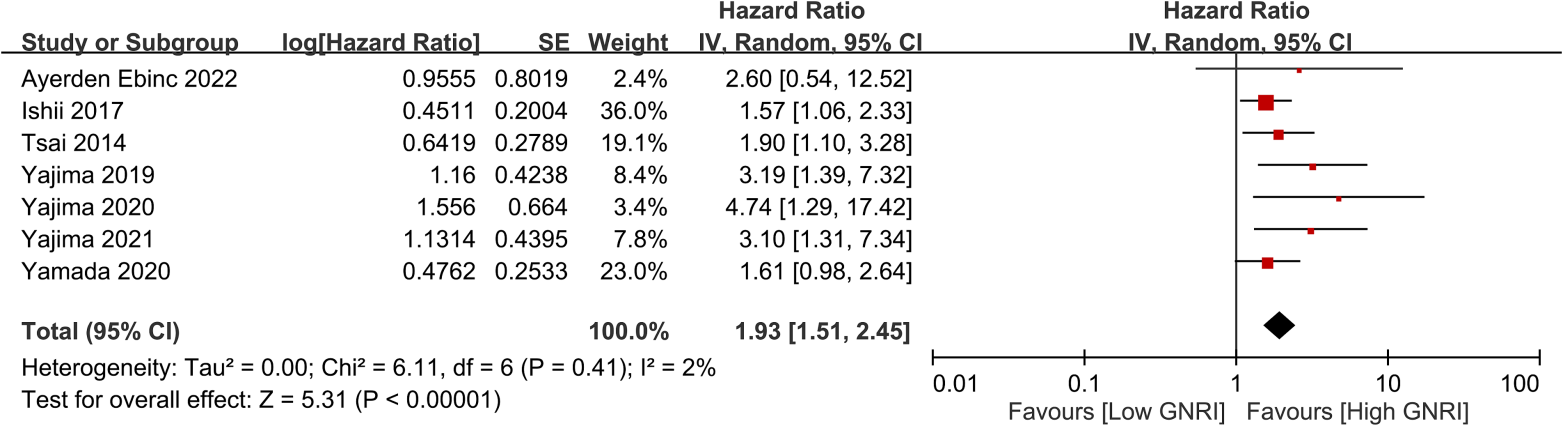
Forest plot illustrating the association between the geriatric nutritional risk index (GNRI) and cardiovascular mortality. CI, confidence intervals.

**Figure 11:**
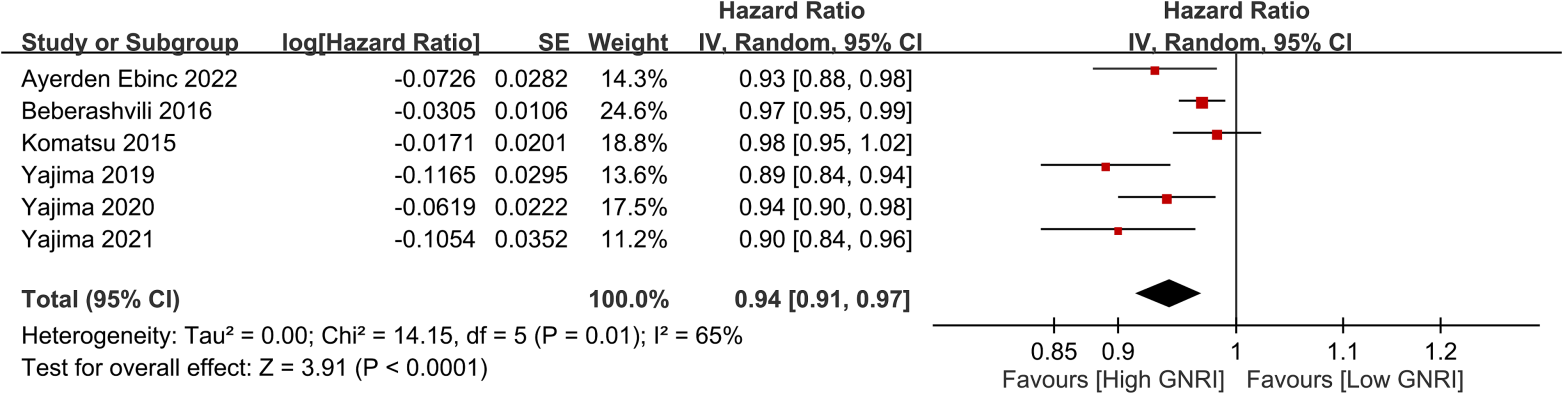
Forest plot showing that with each unit increase in Geriatric Nutritional Risk Index (GNRI), there was a corresponding decrease in cardiovascular mortality risk. CI, confidence intervals.

Meta-analysis on cardiovascular mortality among hemodialysis patients revealed a significant association between lower GNRI scores and increased cardiovascular mortality risk, with a combined HR of 1.93 (95% CI, 1.51–2.45, *p* < 0.00001, *I*^2^ = 2%) (Figure 10) (25–27, 35, 41, 51, 53). The sensitivity analysis revealed a consistent finding using the leave-one-out approach. Similarly, an inverse association was observed between GNRI values and cardiovascular mortality risk (HR: 0.94, 95% CI: 0.91–0.97, *p* < 0.0001, *I*^2^ = 65%) (per unit increase) (Figure 11) (25, 27, 35, 37, 44, 53).

## 4 Discussion

Assessment of the prognostic value of the GNRI can facilitate evidence-based practices to optimize the delivery of nutritional interventions and improve risk stratification for targeted care in high-risk hemodialysis populations. In the meta-analysis of 30 studies involving a total of 55,864 patients, a notable correlation was observed between a lower GNRI and an increase in overall mortality risk (HR: 2.42). Furthermore, it was found that each increment in GNRI corresponded with a reduced overall mortality risk (HR: 0.95). The funnel plot showed negligible publication bias. Subgroup analyses have revealed that the GNRI consistently predicts overall mortality risk across diverse ethnic groups. In addition, meta-regression analyses revealed that variables such as patient age, male proportion, percentage of patients with DM, and follow-up duration did not significantly affect the association between the GNRI and overall mortality risk. In relation to cardiovascular mortality among hemodialysis patients, a significant association was observed between lower GNRI scores and an increased risk (HR: 1.93). Similarly, it was found that each increment in GNRI corresponded with reduced cardiovascular mortality risk (HR: 0.94).

Our findings indicate a significant association between lower GNRI scores and increased overall mortality risk. Having a simple and objective tool for identifying patients at a greater risk of mortality based on their nutritional status is of great clinical value for the following reasons. First, earlier nutritional interventions in patients on hemodialysis may improve outcomes (54, 55). The use of the GNRI as a screening tool may help effectively identify these high-risk patients and enable the implementation of preventive strategies. Second, the association between the GNRI and overall mortality risk contributes to better-informed discussions with patients regarding their expected health outcomes. However, the analyzed studies were observational; therefore, causal conclusions cannot be drawn regarding the effect of nutritional status improvements on mortality. Nevertheless, the consistency of the association, despite the population differences, supports the reliability of the findings. Overall, this meta-analysis provides robust evidence that the GNRI could be a practical nutritional assessment tool that can be incorporated into the care of hemodialysis patients globally to identify those with a higher mortality risk. Additional studies are warranted to determine the effects of nutritional support guided by GNRI scores on patient-centered outcomes.

An important strength of this meta-analysis was the inclusion of data solely from patients undergoing hemodialysis. Patients receiving other forms of dialysis such as peritoneal dialysis were excluded. Combining data across different dialysis modalities could potentially impact mortality outcomes (56) and limit the generalizability of the findings to specific populations. By focusing our meta-analysis specifically on patients receiving hemodialysis, we were able to evaluate the GNRI-mortality association in a distinct patient group with shared characteristics. Compared with a previous meta-analysis of 19 studies involving 10,739 patients (22), the present analysis significantly expands the evidence base, encompassing 30 studies with a cumulative total of 55,864 patients. The increase in sample size enhanced the statistical robustness and confidence in the outcomes. Furthermore, our analysis benefits from the incorporation of studies published within a more contemporary window from 2010 to 2023, providing insights from data collected over the last decade, whereas the previous analysis included studies published up to 2018 (22). Analytically, our meta-analysis advances by using time-to-event data such as HRs, which are more indicative of longitudinal trends in patient health, as opposed to the single point ORs previously used (22). Finally, the robustness of our research findings is bolstered by the thorough methodology used, including meta-regression and subgroup analysis, which adds to the overall strength of our results.

Compared with other clinical predictors, the GNRI may serve as a stronger prognostic factor. A previous meta-analysis of 28 studies revealed that geriatric impairments such as functional and cognitive decline, as well as falls, were associated with a higher mortality risk in elderly dialysis patients (OR: 1.14–1.45), in addition to traditional factors such as age and comorbidities (57). A review of nine cohort studies demonstrated that low handgrip strength was significantly associated with an increased mortality risk in dialysis patients (risk ratio [RR]: 1.88) (58). Furthermore, a meta-analysis of eight observational studies involving 190,163 patients revealed a paradoxical association between low body mass index and a higher mortality risk (RR: 1.22) in dialysis patients (59). Another meta-analysis of 38 studies highlighted serum albumin as having a strong inverse association with mortality in patients on dialysis (HR: 0.7038) (60). Our finding that a lower GNRI is associated with increased overall mortality risk, as indicated by a combined HR of 2.42, highlights the utility of this predictor in clinical settings.

The efficacy of the GNRI in predicting overall mortality may vary across populations. For the general population, a previous meta-analysis of 26 observational studies involving 17,097 participants revealed a significant association between low GNRI and an increased risk of both all-cause (HR: 1.32) and cardiovascular (HR: 2.10) mortalities (61). For elderly patients with heart failure, a meta-analysis of nine studies involving 7,659 patients revealed that low GNRI was predictive of all-cause mortality (HR: 1.56) compared to high GNRI (62). In addition, low GNRI values have been associated with an increased overall mortality risk in patients with head and neck, gastrointestinal, or lung cancer, with HRs ranging from 2.39 2.84 (63–65). Considering our findings and previous research, it appears that using the GNRI as a prognostic tool in patients on hemodialysis or suffering from cancer is more beneficial than in other clinical situations.

Despite the focus of our meta-analysis on studies predominantly from Asian countries, there is evidence that the use of nutritional indices to predict long-term outcomes is equally feasible and effective in non-Asian populations. A recent study that examined 101,616 hemodialysis patients in the United States found that higher Prognostic Nutritional Index (PNI) quartiles were associated with stepwise reductions in all-cause mortality (66). For instance, compared with the lowest PNI quartiles, patients in the highest quartile had a 64% lower mortality (66). Furthermore, a higher PNI predicted mortality better than albumin or lymphocyte count alone (66). A key distinction between the GNRI and PNI is that the former relies on serum albumin, body weight, and ideal weight, whereas the latter incorporates albumin and lymphocyte count. Although both use distinct formulations, recent research (66) and our meta-analysis have demonstrated that nutritional indices are effective in predicting the clinical outcomes of hemodialysis patients regardless of their ethnicity. Compared to the PNI, the simplicity of the GNRI, owing to its exclusion of cell counts, makes it a practical and valuable tool in clinical settings.

This meta-analysis had several limitations that need to be acknowledged. First, all the included studies were observational in nature, which prevented the determination of causal associations between the GNRI and mortality outcomes. Residual confounding factors may still be present despite adjustments made in the analyses of individual studies. Second, there was moderate to substantial heterogeneity among the studies for the primary outcome, indicating variability in effects and populations. We addressed this by using a random-effects model and conducting meta-regression; however, some heterogeneity was likely due to differences in the study design and patient factors that could not be accounted for. Potential sources of heterogeneity include differences in patient characteristics, such as comorbidities and dialysis vintages, variation in ethnicities and healthcare systems across countries, and lack of standardized cut-off values used for categorizing high and low GNRI groups. Third, the majority of studies were conducted in Asian countries, with relatively few focusing on Western populations. Additional research may be warranted to confirm the generalizability of these findings to diverse ethnic groups globally. Finally, publication bias is a concern in meta-analyses, although we did not find evidence of significant bias based on visual inspection of funnel plots. However, studies with small sample sizes and null findings may be underrepresented. Overall, this meta-analysis provides strong evidence for the utility of the GNRI as a prognostic marker in hemodialysis patients; however, the limitations highlight the need for cautious interpretation and further high-quality longitudinal studies.

## 5 Conclusion

This meta-analysis of 30 studies involving 55,864 patients revealed an inverse association of the GNRI with overall and cardiovascular mortalities, highlighting the potential of the GNRI as a predictor of patient outcomes. Consistent findings across ethnicities and the lack of influence of age, sex, DM, and follow-up duration on this association highlight the reliability of the GNRI as a prognostic tool. These results underscore the importance of nutritional assessment in patient care and advocate for future research to explore the GNRI as a guide for nutritional interventions to enhance patient survival.

## Data availability statement

The original contributions presented in the study are included in the article/supplementary material, further inquiries can be directed to the corresponding author.

## Author contributions

K-CH: Conceptualization, Data curation, Formal analysis, Writing – original draft, Writing – review & editing. C-LK: Conceptualization, Data curation, Writing – original draft, Writing – review & editing. C-WH: Investigation, Methodology, Resources, Writing – original draft, Writing – review & editing. C-HY: Methodology, Software, Writing – original draft, Writing – review & editing. C-ML: Methodology, Writing – original draft, Writing – review & editing. H-TC: Conceptualization, Methodology, Writing – original draft, Writing – review & editing. Y-JC: Methodology, Writing – original draft, Writing – review & editing. S-WL: Investigation, Methodology, Writing – original draft, Writing – review & editing. I-WC: Conceptualization, Data curation, Formal analysis, Methodology, Writing – original draft, Writing – review & editing.
